# The Defective and Dependent

**Published:** 1911-11-04

**Authors:** 


### THE DEFECTIVE AND DEPENDENT.

### Introductory.

We propose in this series of articles to deal with certain bodily and mental infirmities, dating from birth or from an early age, which render the unfortunate subjects of them incapable of occupying a normal place in the community into which they are born. In the absence of ameliorative treatment such persons must inevitably be left behind in the march of modern civilisation, and sink into a condition of degradation and permanent dependence. Some of these ameliorative methods and their beneficial results will be described in the course of these papers, and it will be shown how by early and sedulous attention much may be done to improve the condition of those who by reason of original defect are, without such assistance, predestined to become a permanent and oppressive charge upon Society.

### A Classification.

Such cases may be roughly divided into three classes in view of the manifestation of their most prominent symptoms as follows:—

(*a*) Those affecting the intelligence.

(*b*) Those affecting the senses.

(*c*) Those chiefly affecting the muscular and other physical functions.

It must, however, be borne in mind that, while such a classification is convenient for descriptive purposes, a basis of physical defect underlies all the varieties set forth. Cases of mental defect, for example, depend on some defect in the physical development of the brain or of its constituent cells, the result of which is that normal manifestations of mind are interfered with. Similarly, sensorial defects depend upon physical malformations or defects, original or acquired, of the sense organs. The specific class of more purely physical defects with which we propose to deal may be the result of original formative error or of diseases in early childhood.

Under the term mental defect we include all degrees of original failure to develop normal intelligence, though excluding mental breakdown in after-life in the forms which are commonly known as insanity. Mental defectives are commonly grouped into the three gradations of Idiots, Imbeciles, and Feeble-minded in proportion to the more profound or more superficial character of their infirmities.

Under the designation of physically defective we include those handicapped by bodily malformations or paralytic conditions dating from birth or infancy, or by developmental disorders in early childhood. Such are incapacitated by their infirmities from sharing the education of ordinary children and thus form a class apart. As examples of the conditions first referred to we may instance cardiac imperfections and birth-palsies, and of the second, rickets and tuberculous affections of the bones and joints. Epilepsy occurring early in life may also be regarded as a physical ailment, though tending to intellectual enfeeblement and thus linking those so affected with the mentally defective class. While those popularly known as "Cripples" form a large contingent of this category, it may be noted that for administrative purposes the tendency is to include under the term "physically defective" an increasing number of chronic invalids, such as phthisical children who share in the benefits of Open Air Schools recently provided by certain educational authorities.

### Society and Defectives.

The arrangements made by the more enlightened communities of this and other countries for the early recognition and appropriate treatment of the various classes of defectives referred to entail necessarily considerable expense, and it may be well at once to consider the economic aspects of the question. How far, it may be asked, is the cost to the rates of the system in vogue for the special training of the blind and deaf, and the mentally and physically defective, justified by corresponding benefits to the community? Without suitable training in early life it is obvious that the vast majority of defective children will become permanently dependents in adult life, and it is probable that many, especially those of the mentally-defective class, will, if left to themselves, swell the ranks of the delinquents. Thus the State cannot escape its obligation to provide at one or other period of life for its defective members, and it is surely more reasonable, as well as more humane, to expend public funds on their educational improvement in childhood rather than in their maintenance in adult life as paupers or criminals. The special instruction of children suffering from the various defects under consideration is of necessity expensive, as they require to be handled by well-skilled teachers in comparatively small groups. According to recent returns of the Education Officer of the London County Council* it would appear that the average cost of day-school education for the blind and deaf is more than four times that of the ordinary child in the Elementary school, while that of the mentally-defective, and of the physically-defective, respectively, is twice and three times as much. As far as can be judged from tables appended to the same Report relating to the after-career of those who have left the London Special Schools for the several classes of children under consideration it would appear that from 70 to 80 per cent. of those tabulated for four consecutive years after leaving school had found more or less remunerative employment, and that the weekly wages earned by those at work in the fourth year averaged from 7*s.* 11*d.* for the mentally-defective to 12*s.* 9*d.* for the blind. Such figures are interesting as serving to show some monetary return for the large expenditure in training, but of course can only be regarded as of an approximate character.

* See *Report of Education Committee L.C.C.* (Part 2). P. S. King and Son, Westminster (March 1, 1910).

